# Attitudes and Practice Regarding Disposal for Unwanted Medications among Young Adults and Elderly People in China from an Ecopharmacovigilance Perspective

**DOI:** 10.3390/ijerph16081463

**Published:** 2019-04-25

**Authors:** Xiaotan Yu, Xianmin Hu, Shulan Li, Mengya Zhang, Jun Wang

**Affiliations:** 1 New Medicine Innovation and Development Institute, Department of Pharmacy, College of Medicine, Wuhan University of Science and Technology, Wuhan 430065, China; yuxiaotan123@gmail.com (X.Y.); huxianmin@wust.edu.cn (X.H.); lishulan971204@outlook.com (S.L.); zhangmengya0703@163.com (M.Z.); 2 Hubei Province Key Laboratory of Occupational Hazard Identification and Control, Wuhan University of Science and Technology, Wuhan 430065, China

**Keywords:** ecopharmacovigilance, disposal of unwanted medicines, pharmaceutical, environment, young adults, elder people

## Abstract

Due to the expensive cost and uncertain effectiveness of environmental management options in eliminating pharmaceutical residues, recently, decreasing the emission of pharmaceutical pollutants from a drug administration perspective has been considered a hot area of research. As a kind of drug administration for the environment, ecopharmacovigilance (EPV) emphasizes the source control of pharmaceutical pollutants. Disposal of unwanted medicines has been considered as the easiest target for source control of pharmaceutical contamination. Here, we focused on public attitudes and practice regarding disposal of unwanted medicines from the EPV perspective among 365 Chinese university young adults and 206 elderly retirement home residents. The results showed that the majority of respondents had positive attitudes, but exhibited inadequate awareness and poor practice. In addition, the young-adult respondents were found to pay more attention to the environmental problems posed by pharmaceutical residues, and be more supportive of the EPV intervention predominantly performed by pharmaceutical industries and pharmacists. Therefore, it is urgent to establish the standard medicine disposal protocols and educate the general public on the best way for medication disposal under the principle of EPV in China, and efforts on environmentally-preferred drug disposal under EPV should target for the specific demographics.

## 1. Introduction

To date, pharmaceuticals as a kind of emerging contaminant in the natural environment have caused concern among environmental and health researchers because of their ubiquitous occurrence and intrinsic biological/toxicological activities [1,2,3,4]. The presence of a great diversity of commonly used pharmaceuticals, such as antibiotics, anti-inflammatory drugs, antidiabetics, hypotensive drugs, hormones and antidepressants, have been frequently reported to be detected in the environment, including water, sediments, soil, etc., even in the food chain through crop growth and irrigation [3,4,5,6]. Due to their high volume of use, their continuous introduction into the environment, and the lack of effective removal of pharmaceutical residues from the environment, pharmaceuticals have been referred to as pseudo-persistent contaminants [1,7]. As biologically active compounds designed to be effective even at low concentrations, pharmaceuticals that occur in the environment can lead to chronic toxic effects for organisms. In fact, an increasing amount of evidence has shown the negative impacts of pharmaceuticals in the environment on ecosystems, even on human health via indirect exposure. For example, long-term exposure to antibiotic residues in the environment contributes to the development of antibiotic-resistant bacteria and/or genes [4,8]. The veterinary use of a non-steroidal anti-inflammatory drug diclofenac, and subsequent food-chain exposure, trigger the near extinction of several Asian Gyps vulture species [7]. In addition, the presence of 17α-ethynylestradiol, a widely used estrogen in birth-control pills, in lake water at trace concentrations (5–6 ng/L) has been demonstrated to cause feminization and the near extinction of some fish species from the studied lake [9]. Furthermore, the exposure of school children to veterinary antibiotic residues in contaminated food or water environment was considered to be associated with an increased risk of overweight and obesity [10].

In the past decade, researchers in different fields including environics, management science and pharmacy, etc., have made good progress in the minimization of the environmental load and ecological risks of pharmaceutical residues [11,12,13]. However, due to the expensive cost and uncertain effectiveness of environmental management options in eliminating pharmaceutical residues, recently, decreasing the emission from the source of pharmaceutical pollutants from a drug administration perspective has been considered a hot area of research [12,13,14,15]. As a kind of drug administration for the environment, ecopharmacovigilance (EPV) aims to detect, assess, understand and prevent the adverse effects or other problems related to the presence of pharmaceuticals in the environment, through joining interventions at health and regulatory levels [13,14]. In particular, the control of pharmaceutical pollution sources is the primary focus of EPV activities [7,16]. However, to date, the source management approaches under EPV are still needed to be embodied in order to effectively translate EPV theory into practice.

The commonly recognized sources of pharmaceuticals pouring into the environment include excretion by humans and animals following normal use of medicines, hospitals, pharmaceutical production facilities, aquaculture and livestock, etc. [12,13,17]. Importantly, owing to the population’s huge consumption of over-the-counter medications, the importance of public practices regarding disposal of unwanted medicines as sources of entrance of pharmaceuticals into the environment has been pointed out, and the disposal of leftover medicines that have expired or are unused is considered as the easiest target for source control of pharmaceutical contamination [18,19,20]. Glassmeyer et al. [15] have also proposed that practicing prudent disposal of unwanted medications should be an obvious starting point of reducing the introduction of pharmaceutical residues to the environment.

In order to minimize the entrance of pharmaceuticals into the environment via disposal of unwanted medications, to provide the baseline information needed for future EPV implementation, and to boost the effective source control from an EPV perspective, it is necessary to determine the current situation regarding pharmaceutical disposal behaviors of the public. Based on the theory of planned behavior [21], for the public, positive attitudes toward EPV and environment-friendly pharmaceutical disposal behaviors are an important prerequisite to the acceptance and subsequent behavioral revision, therefore could enhance the effectiveness of future EPV practice. Significantly, it has been proposed that the populations from different demographics appear to vary in their pharmaceutical disposal behaviors, therefore, identifying differences in behaviors is helpful for determining where and how to focus resources and efforts for controlling the various sources of pharmaceutical pollution [19,22].

There have been some published studies undertaken on disposal practices [15,18,19,20,22,23,24]. However, these previous studies have only involved a small number of countries and areas. Moreover, no study has yet combined the pharmaceutical disposal survey and the new EPV theory. China is a populous country where disposal practices of medicines may have a huge impact on the global environment. Therefore, the present study was carried out among Chinese university students and retirement home residents to assess their attitudes and practice regarding disposal for unwanted medications from an EPV perspective. In addition, the findings can provide detailed insights into the public’s attitudes and practice toward EPV-directed pharmaceutical disposal management, present a comparison for the pharmaceutical disposal trends of young adults and elderly people, and offer key insights into how to direct targeted efforts on environmentally-preferred drug disposal practices for the specific demographics under EPV. 

## 2. Materials and Methods 

This was a questionnaire-based, cross-sectional study, conducted over a period of three months from March to May, 2018. The study was approved by the Ethics Committee of Wuhan University of Science and Technology (Project number: WUST-18305). The results combine two surveys in Wuhan University of Science and Technology, a provincial university having about 20,000 students on campus, and in five retirement homes having about 800 elderly people in total, respectively. Students in the university and residents in retirement homes were randomly approached and invited to participate. Student participants were asked to complete the questionnaire themselves, and the elderly people provided their responses marked by the researchers. The elderly retirement home residents with dementia, aphasia or any psychiatric diagnosis were eliminated from the survey.

The initial draft of the questionnaire was developed based on published studies on disposal practices [15,18,19,20,22,23,24] as well as information about EPV [7,13,14,16]. A pharmacy researcher and a public health researcher were asked to appraise the content clarity, relevance, validity and conciseness of the items in the questionnaire. Moreover, pretesting of the survey questionnaire was done on a convenient sample, including 20 students and 20 elderly people who did not participate in the final study. The overall Cronbach’s alpha value was obtained as 0.803. A final questionnaire consisted of 23 structured questions organized into three sections. The first section consisted of four questions about socio-demographic indicators, including age, gender, education and professional backgrounds. The second section requested responses regarding the perception and attitudes concerning pharmaceutical pollution in the environment, the potential environmental and health hazards of pharmaceutical disposal behaviors, as well as EPV-related pharmacy administrative intervention. The responses of these questions were based on a 5-point Likert-scale format: strongly disagree (1), disagree (2), undecided (3), agree (4) and strongly agree (5). The last section of the survey included elements designed to evaluate the actual practices regarding the disposal of pharmaceutical wastes. The final questionnaire was administered to 392 university students and 206 retirement home residents who were willing to take part in the survey after obtaining written informed consent. The original language for this questionnaire was English. The questionnaire was translated into Chinese and back translated into English in order to avoid the misinterpretation.

Data from the surveys were entered into SPSS 20.0 (SPSS Inc., Chicago, IL, USA) for analysis. Results for categorical and continuous variables were respectively presented as numbers (percentages) and mean ± standard deviation (SD). Continuous variables were analyzed and compared using the one-way ANOVA with post hoc Tukey’s HSD test. Chi-square test was used to determine the relationship between the categorical data. Differences were considered to be statistically significant when the *p*-value was ≤0.05.

## 3. Results

### 3.1. Descriptive Statistics

By the end of the surveys, 365 university students and 206 retirement home residents had completed the questionnaire, yielding an overall effective response rate of 95%. The summary of the socio-demographic information of participants was shown in Table 1. The gender and profession distributions of survey respondents were approximately equal between young-adult and elder samples. The age ranges of young-adult and elderly respondents were between 17 and 24 years, between 64 and 92 years, respectively.

### 3.2. Attitudes and Perception Concerning Disposal for Unwanted Medications from an EPV Perspective 

As shown in Table 2, the overall attitudes and perception of all participants concerning disposal for unwanted medications from an EPV perspective were positive. The necessity for proper disposal of unwanted medications was endorsed by 97% of a total of 571 participants. In view of evidence that the concern of the adverse environmental impacts associated with improper disposal is the key motivation for getting rid of unwanted medicines in an environmentally appropriate manner [18,19], the respondents were then asked about the reasons why proper disposal of unwanted medicines is necessary. The results showed that 63%, 66%, 45% and 59% of respondents, respectively, believed it to be to prevent illegal, unintended, intended applications and the environmental pollution posed by pharmaceutical residues. Furthermore, most respondents (79%, 82% and 86%, respectively) agreed or strongly agreed the entrance of pharmaceuticals into environment via disposal of unwanted medications, the potential adverse effects of pharmaceutical residues on the ecosystem, wildlife species, even human beings, and the necessity of pharmaceutical discharge control. 

However, as for the environment-friendly and safe route to dispose of unwanted medications, it was interesting that 48%, 33% and 49% of respondents respectively felt undecided about the environmental safety of commonly used disposal practices for unwanted medications include flushing or washing down the sink or toilet, discarding as solid waste, and returning to a pharmacy take-back system, suggesting a considerable portion of respondents did not know how they can properly dispose of the unwanted medications in order to minimize the entrance of pharmaceuticals into the environment. Then, the respondents were asked about those responsible for creating awareness for proper disposal of unwanted medicines; in turn, pharmacist, pharmaceutical industries, government, public and physicians were believed to bear important responsibilities, which were agreed by 80%, 70%, 58%, 53% and 50% respondents, respectively. This finding indicated that, in the eyes of the public, pharmacy professionals should be proactive about educating on how to dispose of unwanted medications in a more environmentally acceptable manner. 

In addition, the importance of pharmacy administration from the pollution sources was agreed or strongly agreed by 86% of participants in the study. When examining respondents’ willingness to participate in any future EPV-related practices, it was encouraging to find that the pharmacy administrative intervention emphasizing “source control” of pharmaceutical pollution, as well as the provably safe and environment-friendly route to dispose of unwanted medications were endorsed and adhered to by 93% and 82% of respondents, respectively. Accordingly, 87% of respondents stated that they want to obtain the information and knowledge about potential environmental risks of pharmaceutical residues, rational disposal, take-back and management of unwanted medications.

The comparison of perception and attitudes concerning disposal for unwanted medications from an EPV perspective between young adults and elderly people is shown in Table 2. In general, the elderly people showed more positive attitudes and perception than the young adults. However, the full recognition of the necessity for proper disposal of unwanted medications (Q1) and the fundamental role of pharmacy administration in solving the environmental problems posed by pharmaceutical residues (Q10), the lack of perception of the pharmacy take-back system (Q8), the high positive willingness to accept the provably safe/environment-friendly route to dispose of unwanted medications (Q12) as well as the information and knowledge about potential environmental risks of pharmaceutical residues, rational disposal, take-back and management of unwanted medications (Q13) were comparable between two samples. Moreover, the young adults appeared to be more aware of “to prevent the environmental pollution posed by pharmaceutical residues” as the reason why proper disposal of unwanted medicines is necessary (Q2, *p* < 0.05), and have a more favorable attitude for the pharmacy administrative intervention emphasizing “source control” of pharmaceutical pollution (Q11, *p* < 0.01) than the elderly people. In addition, there was difference in the response to the question “who should be responsible to create awareness for proper disposal of unwanted medicines?” between young adults and elderly people. The young adults tend to think that pharmaceutical industries and pharmacists should be more responsible to create awareness for proper disposal of unwanted medicine; but the elderly people put more focus on the roles of government and physicians.

### 3.3. Disposal Practice for Unused Medicines

**Figure 1:**
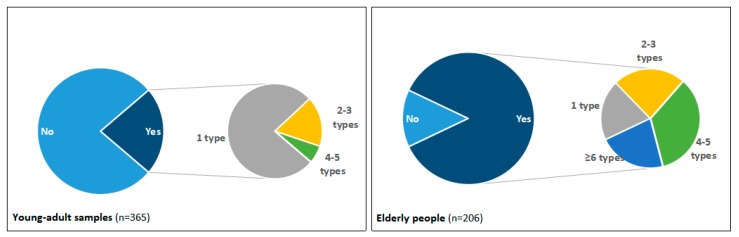
Graph showing whether and how many unused medicines respondents keep in their homes in young-adult and elderly samples.

**Figure 2:**
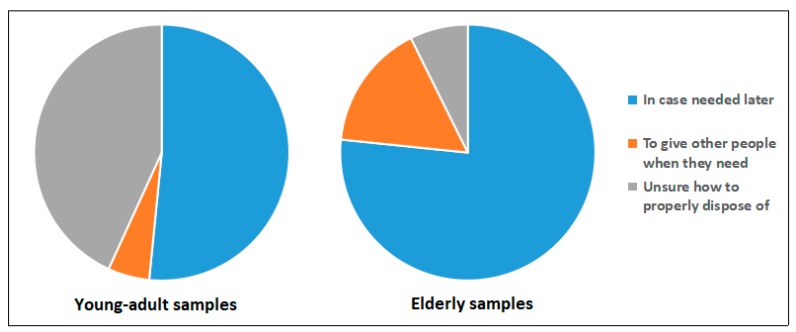
Graph showing why respondents keep unused medicines in young-adult and elderly samples. Respondents (*n* = 82 and 177 in young-adult and elderly samples, respectively) providing one or more reasons (*n* = 95 and 231 answers in young-adult and elderly samples, respectively).

**Figure 3:**
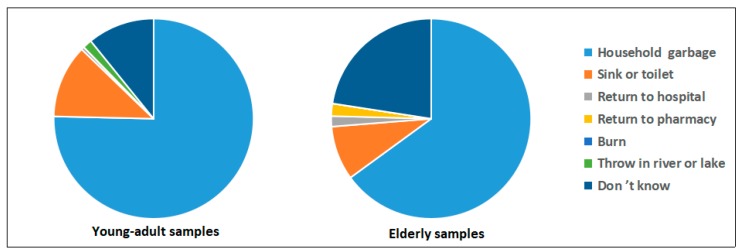
Graph showing preferred method of medication disposal in young-adult and elderly samples. Respondents (*n* = 365 and 206 in young-adult and elderly samples, respectively) providing one or more methods of medication disposal (*n* = 406 and 297 answers in young-adult and elderly samples, respectively).

**Figure 4:**
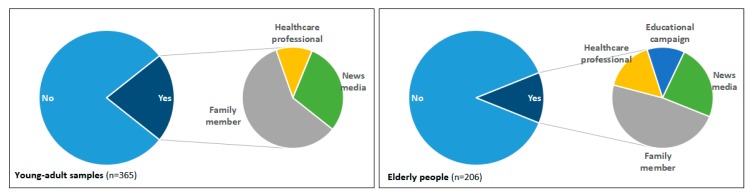
Graph showing whether and how respondents have been given advice on medication disposal in young-adult and elderly samples.

As shown in Figure 1, Figure 2, Figure 3 and Figure 4, questions about the current disposal practice for medicines were provided to the young-adult and elderly samples. When asked “Now, do you have medicines in your home?”, 22% of university students and 86% of elderly people answered yes, and significantly more elderly people preferred to keep medicines in their homes (χ^2^ = 213.9, *p* < 0.01). Among those having medicines in their home, a considerable number of students (77%) only had one type of medicine, this proportion was substantially lower in elderly people (20%), but 80% of elderly people keeping medicines had more than two types of medicine. The most often chosen reason for keeping medicines was “in case needed later,” which was agreed on by 49 (60%) students and all (100%) elderly people having medicines in their homes. Moreover, 50% (41) students having medicines in their homes stated that “I am not sure how to properly dispose of these unused medicines,” compared to 10% (17) of elderly respondents, suggesting more young adults paid attention to the disposal method selection of unused medicines (χ^2^ = 52.6, *p* < 0.01). Then, the respondents were asked the question “If you have ever disposed of unused medicines, how did you dispose of them? If you have not ever disposed of unused medicines, how would you dispose of them?”. Both young-adult and elderly respondents tend to throw unused medicines away in household garbage (84% and 94%, respectively). However, only two students and 11 elderly people stated they disposed of or wanted to dispose of unused medicines to medical stores or pharmacies, and a proportion of respondents (12% of young adults and 33% of elderly people) reported that they did not know how to dispose of unused medicines in their homes, suggesting the actual practice of EPV-directed disposal of unused medicines was poor, and there is still room for improvement. In addition, whether the related advice has been given was reported to be the most influential factor determining whether someone could appropriately dispose of their unused medicines [18]. Here, a majority of respondents (79% of young adults and 88% of elderly people) reported that they had not received advice on how to deal with unused medicines. To either young adults or aged people, family members such as parents and children appeared to be the main advisors on medication disposal.

## 4. Discussion

The environmentally inappropriate disposal of pharmaceuticals in unaltered forms has been well accepted as one of main pollution pathways of pharmaceutical residues in the environment [18,19]. In fact, household medicine storage is a common practice around the world, and a considerable amount of unused medicines were reported to be kept at households due to recovery from disease, expiry, alteration of dosage, adverse effects, promotional practices by manufacturers, physicians’ prescribing practices, patient deaths as well as changes in medicine, etc. [24,25]. In a survey in European countries [23], 88% of the population failed to completely consume medications and kept them at their homes. It can be speculated that, if these unused medications are inappropriately disposed of, a high environmental load of pharmaceutical residues would be generated; therefore, enough attention should be paid to this kind of pollution source. In order to minimize the contamination by pharmaceutical residues, it is crucial to adopt effective pharmacy administrative intervention in normalizing the unused medicine disposal behaviors in the public. As EPV emphasizes “source control” of pharmaceutical pollution, the development of an environment-friendly disposal system of unused medicines should be an indispensable part of EPV protocols [3,13]. In addition, compared with the removal of excreted pharmaceuticals from human and animals’ feces and urine, another identified main source of pharmaceutical pollution, the issue of appropriate medicine disposal should generally be much easier to address [18]. 

In order to explore the appropriate medicine disposal strategies for EPV that would be accepted by the public, in the present study, we evaluated the young adults and elderly peoples’ current attitudes toward unwanted medication disposal from an EPV perspective. The overall attitudes and perception of all 571 Chinese participants concerning disposal for unwanted medications from an EPV perspective were positive. This finding is encouraging, and the positive attitudes can result in active participation of the public in the EPV-directed environment-friendly disposal of unused medicines, when translated into behavior. Environmental awareness is the key to compliance with environment-friendly disposal practices of unused medicines [18,19,20]. Tong et al. [19] mentioned that, when patients were aware of the potential adverse impacts of pharmaceuticals on the environment, they were more likely to return unused medicines in a manner that reduced environmental pollution (i.e., to pharmacies). Here, we found that, despite the fact that the necessity for proper disposal of unwanted medications was well accepted by most respondents, only about half (59%) were concerned about the environmental risks associated with pharmaceutical residues, suggesting the education and awareness building are required for the highlighting of the potential environmental risks associated with inappropriately disposed medicines and the subsequent development of EPV-directed environment-friendly medicine disposal habits. 

Nevertheless, the entrance of pharmaceuticals into the environment via disposal of unwanted medications, the potential adverse environmental effects of pharmaceutical residues, as well as the necessity of pharmaceutical discharge control were agreed by a majority of respondents, which indicated that, when prompted, the public could generate concern over the environmental implication based on their understanding of the intrinsic nature of pharmaceuticals, and perceive this environmental problem to be an urgent issue. Additionally, most respondents wanted to obtain EPV-related information and knowledge. These data showed the high acceptance of EPV-directed environment-friendly medicine disposal among the public. In addition, pharmacists and pharmaceutical industries were mostly considered as the main-bodies responsible for creating awareness for proper disposal of unwanted medicines, which is in line with the finding that most respondents agreed on the importance of pharmacy administration from the pollution sources. Therefore, it could be speculated that, as a pharmacy administrative intervention emphasizing “source control” of pharmaceutical pollution [7,16], EPV as well as disposal approaches of unused medicines advocated under EPV might be well accepted by the public. 

For the public, the commonly used disposal practices for unwanted medications include flushing or washing down the sink or toilet, discarding as solid waste, or returning to a pharmacy take-back system [15,20]. In fact, the consensus on the optimal approach to disposal of unused medications is still lacking, which might be due to the fact that multiple factors such as human health risks (including suicide prevention), environmental considerations, costs or practical considerations [15,23] need to be considered. However, from the perspective of the environment, medicine disposal practices by sewerage will increase the load of pharmaceutical residues to wastewater systems, which are generally designed to remove traditional pollutants (e.g., particulate matter, pathogens, nutrients), not trace concentrations of emerging contaminants such as pharmaceuticals. Additionally, medicine disposal within the sewage system has been considered to be likely to have the greatest impact on water bodies [23]. Disposal via trash could increase the accidental exposure, when being an accessible temptation for pets, children and wildlife. Moreover, unwanted medicines in solid waste will migrate into the landfill leachates, sequentially contaminate the surface and ground waters through the hydrological cycle [20,26]. For example, Paíga et al. [26] have pointed out the importance of wastewater treatment plants, hospitals and landfills as sources of entrance of pharmaceuticals into the environment, and landfill leachates have been demonstrated to account for the highest contribution of the loading of ibuprofen, an anti-inflammatory drug, into surface waters [27]. By contrast, returning to a pharmacy take-back system is attractive as an environmentally appropriate method to dispose of unwanted medications [15,20,27]. 

In the present study, we found much confusion existing about the proper way in which unwanted medicines should be disposed of, which has been also reported in many previous studies [18,19,23]. Of interest is that returning to a pharmacy take-back system, the environment-friendly and safe route to dispose of unwanted medications, was agreed or strongly agreed on only by less than half (46%) of respondents. Accordingly, when asked about the reasons for keeping medicine, 58 of 259 (22%) respondents having medicines in their homes stated that “I am not sure how to properly dispose of these unused medicines,” suggesting the dearth of information about proper medicine disposal reaching the general public. In particular, even if 46% of respondents perceived returning to a pharmacy take-back system as an environment-friendly and safe route to dispose of unwanted medications, it is of concern that only a very small proportion (2%) of respondents currently have the actual practice of selecting the pharmaceutical take-back programs as the means of disposal. Most people tend to throw unused medicines away in household garbage, which was the most convenient and primary disposal approach reported previously [19,20]. 

In addition to the weak environmental awareness, another important reason for the poor practice regarding proper medication disposal is that, in China, the pharmacy take-back service is still in the infant stage. Despite the fact that many Chinese pharmaceutical firms and local governments have begun to sponsor the drug take-back events, there is still no national pharmacy take-back programme or regulation. In fact, the household expired medicines have been added into the National Hazardous Waste List in China [28], however, the current Pharmaceutical Administration Law of the People’s Republic of China has not yet included the provisions on the drug take-back [29]. Accordingly, the supporting take-back sites for proper pharmaceutical disposal have not been well established and available. In such a situation, it is reasonable that the public in China felt undecided about the best way for medication disposal, and rarely returned their unused medicines to the pharmacy take-back system. In fact, pharmaceutical take-back programs are not available and standardized enough in the most countries of the world, especially in developing countries [15,20,27]. The absence or lack of a pharmacy take-back system in Kingdom of Saudi Arabia, Qatar, Bangladesh, Oman, India and Nigeria, etc., has been considered as a key cause of improper medicine disposal behaviors among the general public [30]. A survey [31] conducted in Ghana, a developing country in Africa, showed that, although a comprehensive pharmacy take-back programme named DUMP (Disposal of Unused Medicines Program) had been started, less than 4% of the public respondents disposed of unused medicines by returning them back to the place of purchase, and more than 80% of respondents had never heard about any pharmacy take-back programme in the country. Therefore, it appears that the pharmacy take-back system, as an environmental appropriate disposal route for unused medicine, has more room to be improved in developing countries including China. Even in Europe with widespread standardized take-back programs, not all unused drugs are diverted from the waste stream [18,27]. According to a survey from Germany’s Management Strategies for Pharmaceutical Residues in Drinking Water research program [27], only about a third of the population reported always returning their unwanted drugs to a pharmacy.

Therefore, in order to promote the EPV practice and rational disposal of medicines from an EPV perspective among the general public, it is urgent to enforce the take-back legislation for unwanted medicines, operate the construction of a formalized system of disposal, establish the standard medicine disposal protocols and educate the general public on the best way for medication disposal. The first of these is that public awareness should be raised regarding the need for EPV-directed environmentally appropriate disposal and the routes of disposal. As for the education approach in awareness building, we found that most respondents, regardless of age, considered family members (e.g., parents and children) as the main advisors on medication disposal, suggesting that an initial awareness education campaign targeted towards the general public might be appropriate.

In this study, we compared the attitudes and practice regarding disposal for unwanted medications from an EPV perspective between young adults under 25 years of age and elderly people over 60 years old (Table 3). In addition to the similarities between the two studied samples which have been discussed above, we found that respondents differed slightly in their attitudes and practice depending on the age. In general, the elderly people showed more positive attitudes and perception concerning disposal for unwanted medications from an EPV perspective than the young adults, which was consistent with the opinion that the young-adult population has not yet received much attention with regard to the pharmaceutical-related behaviors [22]. In addition, it can be concluded that, the young adults tend to pay more attention to the environmental problems posed by pharmaceutical residues, and appreciate the pharmacy administrative interventions (such as EPV) predominantly performed by pharmaceutical industries and pharmacists. The possible reasons might be that the young are relatively more open to the new ideas and information on the environment problems and emerging contaminants, which can be easily accessed from newspapers, magazines, the internet, TV and radio, etc., in the situation that environmental pollution is increasingly serious. As the young adults determine the future of pharmaceutical disposal and pollution [22], this finding seemed to be encouraging as it indicates that EPV would be well accepted by the public as a tool to manage the environmental problems caused by pharmaceutical residues over the coming decades. Whereas, the elderly people are more conservative, and more accustomed to the traditional government-centered management and the physician-directed educational campaigns.

However, it should be noteworthy that the elderly people usually keep a large volume of unused drugs in their homes, that may require appropriate disposal. The recent global increase in the use of pharmaceutical products has been considered to be related in part to the ageing population and the significant burden of chronic diseases [18,25]. Therefore, more household medicine storage found in ageing respondents can be explained by their higher levels of medication intake for the management of long term degenerative disorders. Therefore, based on understanding of the demographic variations in awareness and practice, developing targeted education campaigns and managements of medicine disposal behaviors of the ageing population might contribute to the achievement of EPV. 

The limitations of this study include its cross-sectional nature and the random “hit and miss” approach of selecting respondents, which might cause some biases. In addition, the young adults were represented by a university student population, which led to the educational levels of two studied samples being incomparable. In addition, only two segments of the population including young adults and elderly people were included in this survey. In order to effectively develop the EPV campaigns to promote good practice concerning disposal for unwanted medications among the public, further studies involving a greater part of the population are needed.

## 5. Conclusions

This study assessing the attitudes and practice regarding disposal for unwanted medications from an EPV perspective among 365 Chinese university young adults and 206 elderly retirement home residents suggested that the majority had the positive attitudes, but exhibited inadequate awareness and poor practice. The necessity for proper disposal of unwanted medications was endorsed by 97% of participants. The environmental pollution posed by pharmaceutical residues was considered as a reason why proper disposal of unwanted medicines is necessary by 59% of respondents. Most respondents agreed on the entrance of pharmaceuticals into the environment via disposal of unwanted medications and the necessity of pharmaceutical discharge control, and were worried about the potential adverse effects of pharmaceutical residues on the ecosystem, and wildlife species, even human beings. However, there is little awareness among the public regarding appropriate routes to dispose of unwanted medicines. Moreover, pharmacy professionals such as pharmacists and pharmaceutical industries were believed to bear important responsibilities for creating awareness for proper medicine disposal. The importance of pharmacy administration from the pollution sources was agreed by most respondents, suggesting EPV as a pharmacy administrative intervention to reduce pharmaceutical pollution might be well accepted by the public. Accordingly, a majority of respondents declared their willingness to participate in any future EPV-related medicine disposal practice. Additionally, the actual practice of EPV-directed disposal of unused medicines was poor. In addition, the young adult respondents were found to pay more attention to the environmental problems posed by pharmaceutical residues, and be more supportive of the EPV intervention predominantly performed by pharmaceutical industries and pharmacists. Therefore, it is urgent to establish the standard medicine disposal protocols and educate the general public on the best way for medication disposal under the principle of EPV in China, and efforts on environmentally-preferred drug disposal under EPV should target specific demographics.

## Figures and Tables

**Table 1:** Demographic information of participants.

Variables and Categories	Young-Adult Samples (*n* = 365)	Elderly Samples (*n* = 206)
Age, years, mean ± SD	20.49 ± 3.21	72.35 ± 9.48
Gender		
Male, *n* (%)	189 (52)	94 (46)
Female, *n* (%)	176 (48)	112 (54)
Education background		
Illiterate, *n* (%)	0 (0)	9 (4)
Elementary school, *n* (%)	0 (0)	31 (15)
Middle school, *n* (%)	0 (0)	69 (33)
High school, *n* (%)	0 (0)	62 (30)
University, *n* (%)	365 (100)	35 (17)
Profession		
Healthcare professional, *n* (%)	27 (7)	13 (6)
Non-professional personnel, *n* (%)	338 (93)	193 (94)

**Table 2:** The perception and attitude of participants including 365 young adults and 206 elderly people concerning disposal for unwanted medications from an ecopharmacovigilance (EPV) perspective.

Survey Question/Statement	Samples	1. Strongly Disagree	2. Disagree	3. Undecided	4. Agree	5. Strongly Agree	Score
**Q 1:** It is necessary to properly dispose of unwanted medications.	Young adults	0 (0)	0 (0)	16 (4)	96 (26)	253 (69)	4.6 ± 0.6
	Elderly people	0 (0)	0 (0)	3 (1)	65 (32)	138 (67)	4.7 ± 0.5
	Total	0 (0)	0 (0)	19 (3)	161 (28)	391 (68)	4.7 ± 0.5
**Q 2:** The reasons why proper disposal of unwanted medicines is necessary:								
■ To prevent illegal applications (e.g., poisoning).	Young adults	2 (1)	3 (1)	135 (37)	177 (48)	48 (13)	3.7 ± 0.7
	Elderly people	0 (0)	0 (0)	71 (34)	49 (24)	86 (42)	4.1 ± 0.9 **
	Total	2 (0)	3 (1)	206 (36)	226 (40)	134 (23)	3.9 ± 0.8
■ To prevent unintended applications by myself, my family, children or individuals with mental disabilities, pets and wildlife.	Young adults	0 (0)	2 (1)	121 (33)	54 (15)	188 (52)	4.2 ± 0.9
	Elderly people	0 (0)	0 (0)	69 (33)	39 (19)	98 (48)	4.1 ± 0.9
	Total	0 (0)	2 (0)	190 (33)	93 (16)	286 (50)	4.2 ± 0.9
■ To prevent intended applications (e.g., suicide).	Young adults	2 (1)	12 (3)	210 (58)	102 (28)	39 (11)	3.4 ± 0.7
	Elderly people	0 (0)	2 (1)	86 (42)	42 (20)	76 (37)	3.9 ± 0.9 **
	Total	2 (0)	14 (2)	296 (52)	144 (25)	115 (20)	3.6 ± 0.8
■ To prevent the environmental pollution posed by pharmaceutical residues.	Young adults	0 (0)	11 (3)	143 (39)	96 (26)	115 (32)	3.9 ± 0.9
	Elderly people	0 (0)	5 (2)	77 (37)	92 (45)	32 (16)	3.7 ± 0.7 *
	Total	0 (0)	16 (3)	220 (39)	188 (33)	147 (26)	3.8 ± 0.8
**Q 3:** Pharmaceuticals could enter into the environment via disposal of unwanted medications.	Young adults	0 (0)	3 (1)	57 (16)	187 (51)	118 (32)	4.2 ± 0.7
	Elderly people	1 (0)	2 (1)	18 (9)	83 (40)	102 (50)	4.4 ± 0.7 **
	Total	1 (0)	5 (1)	75 (13)	270 (47)	220 (39)	4.2 ± 0.7
**Q 4:** Pharmaceutical residues in environment could cause adverse effects on ecosystem, wildlife species, even human beings.	Young adults	12 (3)	21 (6)	49 (13)	201 (55)	82 (22)	3.9 ± 0.9
	Elderly people	0 (0)	5 (2)	17 (8)	89 (43)	95 (46)	4.3 ± 0.7 **
	Total	12 (2)	26 (5)	66 (12)	290 (51)	177 (31)	4.0 ± 0.9
**Q 5:** It is necessary to minimize the entrance of pharmaceuticals into the environment.	Young adults	5 (1)	26 (7)	38 (10)	155 (42)	141 (39)	4.1 ± 0.9
	Elderly people	0 (0)	2 (1)	10 (5)	97 (47)	97 (47)	4.4 ± 0.6 **
	Total	5 (1)	28 (5)	48 (8)	252 (44)	238 (42)	4.2 ± 0.9
**Q 6:** From an environmental perspective, flushing or washing down sink or toilet is an environment-friendly and safe route to dispose of unwanted medications.	Young adults	189 (52)	46 (13)	48 (13)	30 (8)	52 (14)	2.2 ± 1.5
	Elderly people	7 (3)	18 (9)	129 (63)	27 (13)	25 (12)	3.2 ± 0.9 **
	Total	196 (34)	64 (11)	177 (31)	57 (10)	77 (13)	2.6 ± 1.4
**Q 7:** From an environmental perspective, discarding as solid waste is an environment-friendly and safe route to dispose of unwanted medications.	Young adults	230 (63)	52 (14)	81 (22)	2 (0)	0 (0)	1.6 ± 0.8
	Elderly people	49 (24)	34 (17)	108 (52)	15 (7)	0 (0)	2.4 ± 0.9 **
	Total	279 (49)	86 (15)	189 (33)	17 (3)	0 (0)	1.9 ± 1.0
**Q 8:** From an environmental perspective, returning to a pharmacy take-back system is an environment-friendly and safe route to dispose of unwanted medications.	Young adults	5 (1)	16 (4)	166 (45)	84 (23)	94 (26)	3.7 ± 1.0
	Elderly people	3 (1)	5 (2)	115 (56)	38 (18)	45 (22)	3.6 ± 0.9
	Total	8 (1)	21 (4)	281 (49)	122 (21)	139 (24)	3.6 ± 0.9
**Q 9:** Who should be responsible to create awareness for proper disposal of unwanted medicines?								
■ Government	Young adults	1 (0)	6 (2)	197 (54)	122 (33)	39 (11)	3.5 ± 0.7
	Elderly people	0 (0)	1 (0)	37 (18)	70 (34)	98 (48)	4.3 ± 0.7 **
	Total	1 (0)	7 (1)	234 (41)	192 (34)	137 (24)	3.8 ± 0.8
■ Pharmaceutical industries	Young adults	0 (0)	0 (0)	41 (11)	171 (47)	153 (42)	4.3 ± 0.7
	Elderly people	0 (0)	3 (1)	128 (62)	26 (13)	49 (24)	3.6 ± 0.9 **
	Total	0 (0)	3 (0)	169 (30)	197 (35)	202 (35)	4.0 ± 0.8
■ Physicians	Young adults	8 (2)	9 (2)	241 (66)	65 (18)	42 (12)	3.3 ± 0.8
	Elderly people	1 (0)	5 (2)	24 (12)	89 (43)	87 (42)	4.2 ± 0.8 **
	Total	9 (2)	14 (2)	265 (46)	154 (27)	129 (23)	3.7 ± 0.9
■ Pharmacists	Young adults	0 (0)	0 (0)	29 (8)	156 (43)	180 (49)	4.4 ± 0.6
	Elderly people	0 (0)	1 (0)	84 (41)	61 (30)	60 (29)	3.9 ± 0.8 **
	Total	0 (0)	1 (0)	113 (20)	217 (38)	240 (42)	4.2 ± 0.8
■ Public	Young adults	0 (0)	3 (1)	169 (46)	94 (26)	99 (27)	3.8 ± 0.9
	Elderly people	0 (0)	2 (1)	93 (45)	52 (25)	59 (29)	3.8 ± 0.9
	Total	0 (0)	5 (1)	262 (46)	146 (26)	158 (28)	3.8 ± 0.9
**Q 10:** The pharmacy administration from the pollution sources is the fundamental way to solve the environmental problems posed by pharmaceutical residues, even more important than environmental removal.	Young adults	1 (0)	5 (1)	48 (13)	109 (30)	202 (55)	4.4 ± 0.8
	Elderly people	2 (1)	5 (2)	18 (9)	66 (32)	115 (56)	4.4 ± 0.8
	Total	3 (0)	10 (2)	66 (12)	175 (31)	317 (56)	4.4 ± 0.8
**Q 11:** If there is a pharmacy administrative intervention emphasizing “source control” of pharmaceutical pollution, I would endorse it, and be very pleased to cooperate in its implementation if my participation is needed.	Young adults	1 (0)	3 (1)	17 (5)	132 (36)	212 (58)	4.5 ± 0.7
	Elderly people	0 (0)	2 (1)	15 (7)	96 (47)	93 (45)	4.4 ± 0.7 **
	Total	1 (0)	5 (1)	32 (6)	228 (40)	305 (53)	4.5 ± 0.7
**Q 12:** If there is a provably safe and environment-friendly route to dispose of unwanted medications, I would endorse it, and be very pleased to cooperate in its implementation.	Young adults	0 (0)	1 (0)	75 (21)	107 (29)	182 (50)	4.3 ± 0.8
	Elderly people	0 (0)	0 (0)	29 (14)	67 (33)	110 (53)	4.4 ± 0.7
	Total	0 (0)	1 (0)	104 (18)	174 (30)	292 (51)	4.3 ± 0.8
**Q 13:** I want to obtain the information and knowledge about potential environmental risks of pharmaceutical residues, rational disposal, take-back and management of unwanted medications.	Young adults	0 (0)	5 (1)	35 (10)	186 (51)	139 (38)	4.3 ± 0.7
	Elderly people	0 (0)	1 (0)	31 (15)	71 (34)	103 (50)	4.3 ± 0.7
	Total	0 (0)	6 (1)	66 (12)	257 (45)	242 (42)	4.3 ± 0.7

Data are shown as the number (%) or mean ± SD. * *p* < 0.05, ** *p* < 0.01, compared with the corresponding data of young-adult samples.

**Table 3:** Comparison of attitudes and practice regarding disposal for unwanted medications from an EPV perspective between young adults and elderly people in China.

Similarities/ Differences	Aspect	Item	Young Adults	Elderly People
Similarities	Perception and Attitudes	Necessity to properly dispose of unwanted medications.	Most respondents agreed or strongly agreed.	
		The most recognized reason why proper disposal of unwanted medicines is necessary.	To prevent unintended applications by themselves, their family, children or individuals with mental disabilities, pets and wildlife.	
		From an environmental perspective, returning to a pharmacy take-back system is an environment-friendly and safe route to dispose unwanted medications.	Only few respondents disagreed or strongly disagreed. However, about half of respondents were undecided.	
		The pharmacy administration from the pollution sources as the fundamental way to solve the environmental problems posed by pharmaceutical residues.	Most respondents agreed or strongly agreed.	
		If there is a provably safe and environment-friendly route to dispose of unwanted medications, I would endorse it, and be very pleased to cooperate in its implementation.	Most respondents agreed or strongly agreed.	
		I want to obtain the information and knowledge about potential environmental risks of pharmaceutical residues, rational disposal, take-back and management of unwanted medications.	Most respondents agreed or strongly agreed.	
	Practice	The most often chosen reason for keeping medicine.	In case needed later.	
		The preferred method of medication disposal.	In household garbage.	
		Whether the respondents had been given advice on how to do with unused medicines?	Most respondents had not.	
		The main advisors on the way for medication disposal.	Family members.	
Differences	Perception and Attitudes	The most recognized reason why proper disposal of unwanted medicines is necessary.	To prevent the environmental pollution posed by pharmaceutical residues.	To prevent illegal and intended applications.
		The entrance of pharmaceuticals into the environment via disposal of unwanted medications. The adverse effects of pharmaceutical residues in the environment. The necessity to minimize the entrance of pharmaceuticals into the environment.		More elderly people supported.
		The environment-friendly and safe routes to dispose of unwanted medications.	More elderly people supported that flushing or washing down the sink or toilet, as well as discarding as solid waste are environment-friendly and safe.	
		Who should be responsible for creating awareness for proper disposal of unwanted medicine?	Pharmaceutical industries and pharmacists	Government and physicians
		Attitude for the pharmacy administrative intervention emphasizing “source control” of pharmaceutical pollution.	More favorable.	
	Practice	Whether, and how many, unused medicines are kept in respondents’ homes.	Elderly people preferred to keep medicines in their homes, and more types of medicines were kept by them.	
		I am not sure how to properly dispose of these unused medicines	More young adults agreed.	

## References

[1] Daughton C.G. (2016). Pharmaceuticals and the Environment (PiE): Evolution and impact of the published literature revealed by bibliometric analysis. Sci. Total Environ..

[2] Desbiolles F., Malleret L., Tiliacos C., Wong-Wah-Chung P., Laffont-Schwob I. (2018). Occurrence and ecotoxicological assessment of pharmaceuticals: Is there a risk for the Mediterranean aquatic environment?. Sci. Total Environ..

[3] Wang J., He B., Yan D., Hu X. (2017). Implementing ecopharmacovigilance (EPV) from a pharmacy perspective: A focus on non-steroidal anti-inflammatory drugs. Sci. Total Environ..

[4] Wang J., He B., Hu X. (2015). Human-use antibacterial residues in the natural environment of China: Implication for ecopharmacovigilance. Environ. Monit. Assess..

[5] Voigt M., Savelsberg C., Jaeger M. (2018). Identification of Pharmaceuticals in The Aquatic Environment Using HPLC-ESI-Q-TOF-MS and Elimination of Erythromycin Through Photo-Induced Degradation. J. Vis. Exp..

[6] Kim H.Y., Lee I.S., Oh J.E. (2017). Human and veterinary pharmaceuticals in the marine environment including fish farms in Korea. Sci. Total Environ..

[7] He B.S., Wang J., Liu J., Hu X.M. (2017). Eco-pharmacovigilance of non-steroidal anti-inflammatory drugs: Necessity and opportunities. Chemosphere.

[8] Chen D., Liu S., Zhang M., Li S., Wang J. (2018). Comparison of the occurrence of antibiotic residues in two rural ponds: Implication for ecopharmacovigilance. Environ. Monit. Assess..

[9] Kidd K.A., Blanchfield P.J., Mills K.H., Palace V.P., Evans R.E., Lazorchak J.M., Flick R.W. (2007). Collapse of a fish population after exposure to a synthetic estrogen. Proc. Natl. Acad. Sci. USA.

[10] Wang H., Wang N., Wang B., Fang H., Fu C., Tang C., Jiang F., Zhou Y., He G., Zhao Q. (2016). Antibiotics detected in urines and adipogenesis in school children. Environ. Int..

[11] Munoz M., Mora F.J., de Pedro Z.M., Alvarez-Torrellas S., Casas J.A., Rodriguez J.J. (2017). Application of CWPO to the treatment of pharmaceutical emerging pollutants in different water matrices with a ferromagnetic catalyst. J. Hazard. Mater..

[12] Blair B.D. (2016). Potential Upstream Strategies for the Mitigation of Pharmaceuticals in the Aquatic Environment: A Brief Review. Curr. Environ. Health Rep..

[13] Holm G., Snape J.R., Murray-Smith R., Talbot J., Taylor D., Sörme P. (2013). Implementing ecopharmacovigilance in practice: Challenges and potential opportunities. Drug Saf..

[14] Wang J., Zhang M., Li S., He B. (2018). Adapting and applying common methods used in pharmacovigilance to the environment: A possible starting point for the implementation of eco-pharmacovigilance. Environ. Toxicol. Pharmacol..

[15] Glassmeyer S.T., Hinchey E.K., Boehme S.E., Daughton C.G., Ruhoy I.S., Conerly O., Daniels R.L., Lauer L., McCarthy M., Nettesheim T.G. (2009). Disposal practices for unwanted residential medications in the United States. Environ. Int..

[16] Wang J., Zhao S.Q., Zhang M.Y., He B.S. (2018). Targeted eco-pharmacovigilance for ketoprofen in the environment: Need, strategy and challenge. Chemosphere.

[17] Rodríguez-Navas C., Björklund E., Bak S.A., Hansen M., Krogh K.A., Maya F., Forteza R., Cerdà V. (2013). Pollution pathways of pharmaceutical residues in the aquatic environment on the island of Mallorca, Spain. Arch. Environ. Contam. Toxicol..

[18] Vellinga A., Cormican S., Driscoll J., Furey M., O’Sullivan M., Cormican M. (2014). Public practice regarding disposal of unused medicines in Ireland. Sci. Total Environ..

[19] Tong A.Y., Peake B.M., Braund R. (2011). Disposal practices for unused medications around the world. Environ. Int..

[20] Massoud M.A., Chami G., Al-Hindi M., Alameddine I. (2016). Assessment of Household Disposal of Pharmaceuticals in Lebanon: Management Options to Protect Water Quality and Public Health. Environ. Manage..

[21] Sánchez-Medina A.J., Romero-Quintero L., Sosa-Cabrera S. (2014). Environmental management in small and medium-sized companies: An analysis from the perspective of the theory of planned behavior. PLoS ONE.

[22] Vatovec C., Van Wagoner E., Evans C. (2017). Investigating sources of pharmaceutical pollution: Survey of over-the-counter and prescription medication purchasing, use, and disposal practices among university students. J. Environ. Manage..

[23] Fenech C., Rock L., Nolan K., Morrissey A. (2013). Attitudes towards the use and disposal of unused medications in two European Countries. Waste Manag..

[24] Bashaar M., Thawani V., Hassali M.A., Saleem F. (2017). Disposal practices of unused and expired pharmaceuticals among general public in Kabul. BMC Public Health.

[25] Teni F.S., Surur A.S., Belay A., Wondimsigegn D., Gelayee D.A., Shewamene Z., Legesse B., Birru E.M. (2017). A household survey of medicine storage practices in Gondar town, northwestern Ethiopia. BMC Public Health.

[26] Paíga P., Santos L.H., Amorim C.G., Araújo A.N., Montenegro M.C., Pena A., Delerue-Matos C. (2013). Pilot monitoring study of ibuprofen in surface waters of north of Portugal. Environ. Sci. Pollut. Res. Int..

[27] Lubick N. (2010). Drugs in the environment: Do pharmaceutical take-back programs make a difference?. Environ. Health Perspect..

[28] Ministry of Ecology and Environment of the People’s Republic of China National Hazardous Waste List in China. http://www.mee.gov.cn/gkml/hbb/bl/201606/t20160621_354852.htm.

[29] China Food and Drug Administration Pharmaceutical Administration Law of the People’s Republic of China. http://samr.cfda.gov.cn/WS01/CL0784/124980.html.

[30] Paut Kusturica M., Tomas A., Sabo A. (2017). Disposal of Unused Drugs: Knowledge and Behavior Among People Around the World. Rev. Environ. Contam. Toxicol..

[31] Sasu S., Kümmerer K., Kranert M. (2012). Assessment of pharmaceutical waste management at selected hospitals and homes in Ghana. Waste Manag. Res..

